# Inferring direct regulatory targets from expression and genome location analyses: a comparison of transcription factor deletion and overexpression

**DOI:** 10.1186/1471-2164-7-215

**Published:** 2006-08-22

**Authors:** Lin Tang, Xiao Liu, Neil D Clarke

**Affiliations:** 1 Biophysics and Biophysical Chemistry, Johns Hopkins School of Medicine, Baltimore, MD, USA; 2 Genome Institute of Singapore, Singapore; 3 AviaraDX Inc., 2715 Locker West, Carlsbad, CA, USA; 4 Developmental Biology, Stanford University School of Medicine, Palo Alto, CA, USA

## Abstract

**Background:**

Effects on gene expression due to environmental or genetic changes can be easily measured using microarrays. However, indirect effects on expression can be substantial. The indirect effects of a perturbation need to be distinguished from the direct effects if we are to understand the structure and behavior of regulatory networks.

**Results:**

The most direct way to perturb a transcriptional network is to alter transcription factor activity. Here, for the first time, we compare expression changes and genomic binding in a simple regulon under conditions of both low and high transcription factor activity. Specifically, we assessed the effects on expression and binding due to deletion of the yeast LEU3 transcription factor gene and effects due to elevation of Leu3 activity. Leu3 activity was elevated through overexpression and the introduction of a mutation that renders the protein constitutively active. Genes that are bound and/or regulated by Leu3 under one or both conditions were characterized in terms of their functional annotations and their predicted potential to be bound by Leu3. We also assessed the evolutionary conservation of the predicted binding potential using a novel alignment-independent method. Both perturbations yield genes that are likely to be direct targets of Leu3, including most of the classically defined targets. Additional direct targets are identified by each of the methods. However, experimental and computational criteria suggest that most genes whose expression is affected by the Leu3 genotype are unlikely to be regulated by binding of the protein.

**Conclusion:**

Most genes that are differentially expressed by Leu3 are not direct targets despite the exceptional simplicity of the regulon, and the unusually direct nature of the perturbations investigated. These conclusions are reached through computational analyses that support and extend chromatin immunoprecipitation data on the identities of direct targets. These results have implications for the interpretation of expression experiments, especially in cases for which chromatin immunoprecipitation data are unavailable, incomplete, or ambiguous.

## Background

Transcriptional programs are extremely complicated, and include a great many indirect effects. One of the great challenges in systems biology is to de-convolute complex transcriptional responses to identify the underlying network of direct, transcription-factor mediated control. An important step in that direction has been the development of genome scale chromatin immunoprecipitation assays (ChIP) that map bound transcription factors onto the genome sequence [1,2]. Binding of a transcription factor within a presumptive control region provides evidence that the gene is regulated directly, and the combination of expression analyses and chromatin can be a powerful way of identifying direct targets [3-5]. However, ChIP data may not be sufficient to identify direct targets because genomic binding can be fortuitous and unrelated to gene regulation. There can also be ambiguities in assigning a bound transcription factor to a putative target gene, particularly in higher eukaryotes where regulatory sites can be far away from the affected gene, and can appear 5' to the transcribed sequence, within the sequence, or even 3' to it. Nevertheless, the combination of expression analysis and ChIP localization of bound transcription factors can provide a compelling statistical argument for the enrichment of authentic target genes. The greater the intersection between bound and regulated genes, the greater the confidence that some of these genes are truly direct targets.

The way a regulatory network is perturbed could have a big effect on the ability to identify direct regulatory targets. The less direct the perturbation, the more likely it is that genes will be regulated in some indirect way. Environmental perturbations, for example, could cause signaling events in addition to those that are known and which the experiment was intended to probe. Environmental perturbations can also be complicated by time-dependent changes in binding and expression. For these reasons, the most direct perturbation that can be made to a transcriptional network is to modify genetically the concentration or activity of a transcription factor. Perturbations of this type are aimed directly at the ultimate effector of gene regulation. In addition, genetic perturbations can be propagated for multiple generations before a comparison is made between the baseline condition of the regulatory network (wild-type cell) and its perturbed state (deleted or overexpressed factor). This effectively eliminates kinetic complexities that may otherwise complicate analyses of expression profile differences following an environmental perturbation.

Here, for the first time, we compare expression and binding under conditions of both low and high transcription factor activity. The genes that are bound and/or regulated under these conditions are assessed computationally in terms of Leu3 binding potential, the evolutionary conservation of that binding potential, and the enrichment of functionally related genes. These analyses highlight the utility of both transcription factor deletion and overexpression in defining direct target genes. The combined analysis of deletion and overexpression experiments also points to a broader physiological role for yeast Leu3 than its historically understood role in branched amino acid metabolism.

## Results

### Transcription factor binding and gene regulation at low activity

We begin our analysis by examining published data on genes whose expression is affected by deletion of LEU3 and genes whose promoters are bound by Leu3 protein [6,7]. We refer to these experiments as "low activity" experiments because both involve Leu3 expressed at endogenous levels. For the transcriptome analysis, expression in a wild-type strain was compared to expression in a leu3Δ strain [6]. For the in vivo binding experiment, chromatin immunoprecipitation (ChIP) was performed using an epitope-tagged protein expressed from its normal genomic location [7]. Thus, both sets of experiments involve Leu3 expressed from its endogenous promoter. We also refer to these experiments as "low activity" because Leu3, like many transcription factors, requires an activation signal to be fully functional as a transcriptional regulator. In this case, the activation signal that turns the Leu3 DNA binding protein into a transcriptional activator is the binding of a metabolic intermediate, α-isopropylmalate (α-IPM). It is not clear what fraction of Leu3 is in an activated state under the growth conditions used in the low activity experiments. A comparison of the expression and ChIP datasets has recently been reported by Boer et al. [6]. We present our own evaluation so that a direct comparison can be made to the new high-activity data reported below.

**Figure 1:**
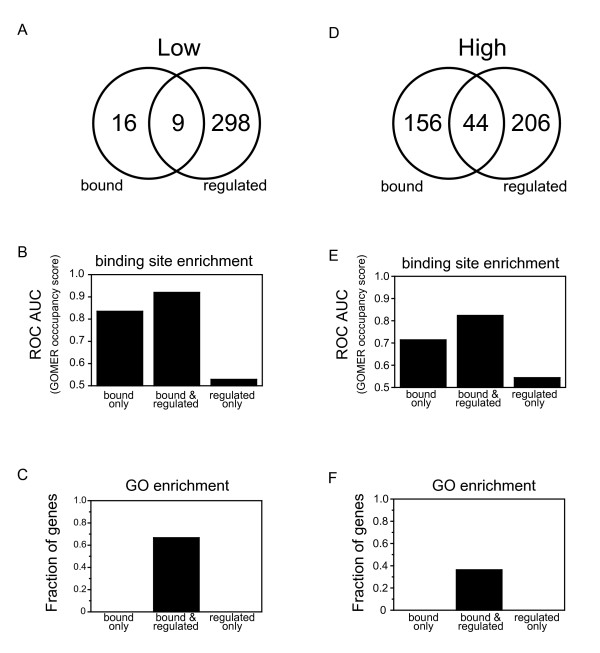
**Comparison of chromatin immunoprecipitation and transcriptome data at low and high Leu3 activity**. (A) Venn diagram showing the number of genes bound and/or regulated at low concentrations. (B) Association of Leu3 binding sites with the indicated subset of genes defined as bound and/or regulated at low concentrations. All genes were scored for predicted Leu3 binding potential using GOMER [8], and ranked according to this score. Genes in the indicated subset were compared to all other genes in the genome using a receiver operator characteristic curve (ROC), and the value of the area under the curve is shown in this histogram. The histogram starts at a value of 0.5 because that is the value expected by chance. A value of 1.0 would indicate that the predicted binding potential scores perfectly discriminate the experimentally defined subset of genes from all others. (C) The fraction of genes in each low concentration subset that have Gene Ontology process annotations that are significantly enriched (P ≤ 1e-6). Gene ontology analyses were performed using the Saccharomyces Genome Database web site [21]. (D–F) These panels are analogous to panels A–C except that the genes were defined based on experiments using constitutively active protein expressed at higher-than-endogenous levels.

Using criteria for Leu3-bound genes [7] and for Leu3-regulated genes [6] as defined by the original authors, only about one third of the genes that are bound by Leu3 are downregulated in a leu3 deletion (Fig 1). The converse is even more striking: only about 3% of the genes whose expression is affected by leu3 deletion are detectably bound by the protein. Boer et al, whose low activity expression data we use here, reached the same conclusion [6].

Why are so many genes affected by leu3 deletion but not bound by the protein? One possibility is that the ChIP experiment is not sensitive enough. To evaluate this possibility we predicted the potential of the regulated genes to be bound by Leu3. If the predicted potential to be bound is high then the failure to detect binding is likely due to insensitivity of the ChIP assay. If, on the other hand, the predicted potential to be bound is as low as random genes, then there is no expectation that these genes should be bound and it is likely that they are regulated indirectly rather than by Leu3 binding.

To calculate the potential to be bound by Leu3, we scored the upstream sequences of all open reading frames (ORFs) in yeast using an equilibrium model for transcription factor binding, implemented in the program GOMER [8], and a position weight matrix (PWM) that we previously defined based on equilibrium dissociation constants for a large number of motif variants [9]. We then ranked all genes by their predicted potential to bind Leu3, and asked whether genes that are bound and/or regulated rank significantly high in this list. As expected, genes whose promoters are bound according to the ChIP experiment are, as a group, enriched in Leu3 binding potential, demonstrating a correlation between predicted binding and observed binding (Fig 1B). This correlation exists both for the genes that are bound and regulated and for the genes that are only bound. In contrast, for the group of genes whose expression is affected by leu3 deletion but which are not detectably bound by Leu3, we find that the predicted Leu3 binding potential is only slightly greater than what is expected by chance. This effect is attributable to a small number of genes with higher-than-average binding potential (data not shown). Thus, some of these unbound but regulated genes may be direct targets of Leu3 but are undetected in the ChIP experiment for reasons of experimental sensitivity. However, most of the unbound genes have binding potentials indistinguishable from unregulated genes, and are therefore likely to be indirect targets of Leu3. A search for over-represented motifs among the unbound but regulated genes failed to find any significant motifs.

Expression changes are clearly not a reliable indicator of direct regulation because most differentially expressed genes are not detectably bound and do not have promoter sequences that suggest they *should *be bound. However, expression experiments add considerable value to the chromatin-IP experiments. First, the intersection of the regulated gene set with the bound set, while fairly small, is statistically significant (P < 1e-5). These genes are more likely to be direct targets than genes that are either bound only or regulated only. Second, genes that are both bound and regulated are highly enriched for genes that have related functions. (Fig 1C). Six of the nine genes that are both bound and regulated have been annotated as being involved in "branched chain family amino acid biosynthesis", an enrichment of several hundred fold (p-value ≤ 3e-15). All six of these genes are directly on the committed pathway to leucine or valine biosynthesis. In contrast, neither the bound-only nor the regulated-only gene sets have any highly significant enrichment of Gene Ontology (GO) annotations (p ≤ 1e-6; see Methods).

### High activity chromatin-IP analysis identifies additional Leu3 targets missed under low-activity conditions

To develop a sense for how transcription factor concentration and activity affects binding and regulation, we performed chromatin immunoprecipitation and expression array experiments using a mutant of Leu3 that is constitutively active (i.e., not dependent on αIPM) (Methods) [10]. This protein was also expressed from a plasmid at levels about 8–40 fold higher than endogenous Leu3 expression (data not shown). We refer to the data obtained with this strain as "high activity" data in distinction to the low activity data described above. In the case of the ChIP experiments, "high activity" means higher-than-endogenous protein concentrations. In the case of expression experiments, "high activity" refers to both the expression level and the mutation conferring constitutive activation function. The protein was also fused to maltose binding protein (MBP) for affinity purification in ChIP experiments. Details of the ChIP experiments have been submitted elsewhere because they were performed in the context of a separate study on the effects of chromatin on DNA binding site selection (XL, Cheol-Koo Lee, Joshua A. Granek, NDC, and Jason D. Lieb; submitted). In this paper we report the results of a transcriptome analysis using this same construct, compare the genes whose expression is activated with the genes that are bound by Leu3p under the same conditions, and compare these high-activity results with those found previously under low-activity conditions.

The genes identified by ChIP under high Leu3 activity conditions are almost perfectly a superset of the genes bound at low activity. Of 25 genes whose upstream regions are bound by Leu3 in the low activity experiments (p ≤ 1e-3), we observed binding of 24 at high activity (p ≤ 1e-4). This attests to the quality of the data. Even at a much more stringent confidence level applied to the high activity data (p ≤ 1e-7), 22 of the 25 genes bound at low activity are still found, plus an additional 137 bound genes. In short, nearly all of the genes deemed to be bound at low Leu3 activity are also bound at high activity. More importantly, there are many additional genes that are bound at the same high level of statistical confidence that are not bound in the low activity ChIP experiment.

The bound genes identified in the high-activity ChIP experiment can be used to identify additional direct target genes that were missed in the low-activity analysis. Amongst the several hundred genes identified as being bound only in the high-activity ChIP experiment the number of genes whose expression is affected by leu3 deletion is about twice as great as the number expected by chance (p < 1e-3 for the null model being no enrichment of leu3Δ-affected genes). This suggests that there are indeed additional direct target genes among the leu3Δ-affected genes that were missed in the low-activity ChIP experiment, perhaps due to insensitivity of the ChIP assay. On the other hand, this is a very modest effect because only a few percent of the leu3Δ-affected genes are bound even under high-activity conditions. This is consistent with the fact that genes affected by leu3 deletion, but which are unbound, tend not to have high predicted Leu3 binding potential.

### Combined expression and chromatin-IP analysis under high-activity conditions

To determine expression levels under conditions of high-activity Leu3, the same constitutively active, plasmid-expressed MBP-Leu3 fusion strain that was used in the ChIP analysis was analyzed in a microarray-based expression experiment. In contrast to the ChIP analyses, which showed that the genes bound at high Leu3 activity include essentially all the genes bound at low-activity, only about 5–10% of genes whose expression is decreased in the leu3 deletion strain are induced by overexpression of constitutively active Leu3 (the exact fraction depends on the criteria used to define differential expression). The small number of genes in common is not surprising if most genes that are differentially expressed are the result of indirect effects.

Different array platforms were used in our high-activity expression and ChIP experiments, requiring different algorithms for the estimation of statistical significance. To define a set of genes that are both bound and induced, we determined threshold p-values for binding and induction that maximized the fraction of genes that meet both criteria, above and beyond the number expected by chance (see Methods). By this criterion, there are 44 genes in common among the top 200 Leu3-bound genes and the top 250 Leu3-induced genes (Fig 1D). These 44 genes are significantly greater than the 9 or 10 that are expected to be in the intersection by chance (p <= 4e-20).

We performed binding-potential and GO-enrichment analyses on the bound, regulated and bound and regulated gene sets, as described above for the low activity data. The trends are the same (Fig 1E,F). Genes that are bound are associated with higher predicted Leu3 binding potential and genes that are both bound and induced are even higher in Leu3 binding potential. The enrichment of binding potential in these genes is lower than for the genes identified in the low activity experiment, but this is expected because the genes bound at low Leu3 concentration are more likely to have more and better Leu3 binding sites than the genes bound at high Leu3 concentrations. As in the low activity analysis, most genes induced by over-expression of constitutively active Leu3 appear to be regulated indirectly rather than by direct binding of the protein because the genes that are induced but not bound have predicted binding potential only slightly greater than that of random genes.

Because overexpression may be a non-physiological perturbation it is possible that the bound and regulated genes identified in this experiment are biologically irrelevant. If that were the case, however, we would not expect these fortuitously expressed genes to share biological functions. It is noteworthy, therefore, that the set of genes bound and induced under these conditions is enriched for certain Gene Ontology annotations (Fig 1F). Indeed, even though the fraction of bound and regulated genes that have significant shared GO process annotations is smaller in these high activity experiments than in the low activity set, the absolute number of genes having GO process annotations in common is higher because there are more genes identified in total (44 vs. 9). Thus, genes identified as possible targets under high activity conditions meet two experimental criteria for direct regulation (binding of the factor and differential regulation due to its perturbation) as well as showing a tendency to share biological functions. We conclude that the high-activity data is probably identifying at least some new authentic target genes.

### Conservation of binding potential supports the existence of direct target genes among bound and regulated genes

**Figure 2:**
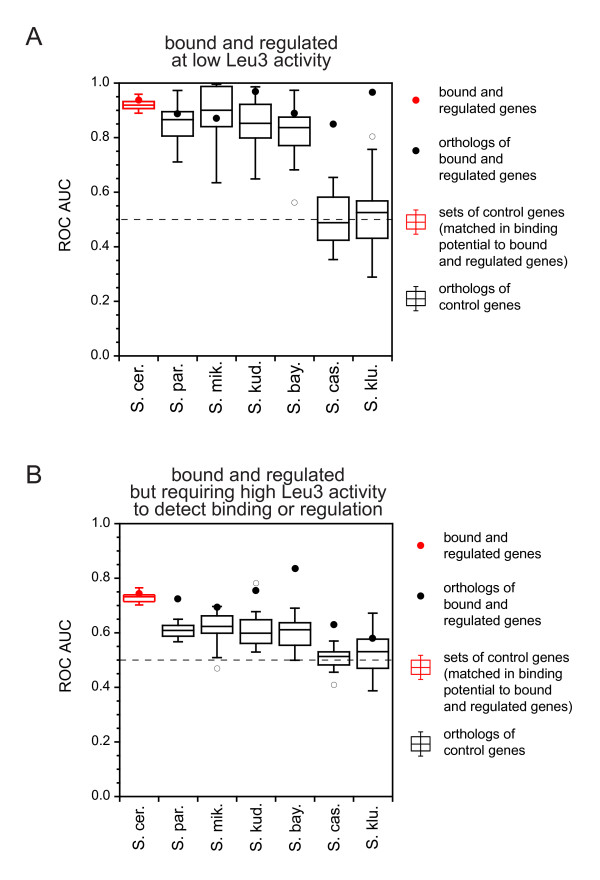
**Bound and regulated genes are more conserved in predicted binding potential than control genes of comparable binding potential**. (A) Predicted binding potential for the 600 bp upstream of every protein-coding gene in S. cerevisiae was calculated using GOMER and a Leu3 position weight matrix, and the calculations were repeated for the 600 bp upstream of all orthologous genes from six other species (Methods). The red circle shows the ROC AUC value obtained from comparing the 9 S. cerevisiae genes that were identified as bound and regulated in low activity experiments with all other genes in the same genome; the black circles are the values calculated for orthologous genes in six related species. The red box plot show the distribution of ROC AUC values obtained from 20 sets of 9 control sequences that were selected to closely approximate the predicted binding potential of the bound and regulated genes (Methods). The black box plots are comparable but are calculated using the orthologs of the genes chosen from S. cerevisiae; the open circles are the box plot outliers. For both experimental and control genes, if an ortholog was undefined in a genome, that gene was simply omitted from the analysis for that genome. Note that the predicted binding potential of bound and regulated genes is maintained in the distantly related species S. castelli and S. kluyveri to a much greater extent than control sequences. (B) Same as panel A except the genes defined as bound and regulated required high Leu3 activity for their detection in either the ChIP or expression assays, or both. There are 45 such genes, 31 of which required high Leu3 activity for detection in both assays. The remainder were detected at low activity in one of the assays (Fig. 3).

Enrichment of GO annotations is one way to evaluate whether a gene set is enriched for biologically relevant targets (Fig 1C,1F). Another is to assess the evolutionary conservation of a gene's predicted binding potential. If genes that are regulated are bound by Leu3 using binding sites that have been selected during evolution, then the promoters of those genes will show evidence of conservation for Leu3 binding. To verify this assumption, we first tested the idea on the nine bound and regulated genes identified in the low activity experiments and evaluated the Leu3 binding potential of their promoters compared to all other genes in the genome. The analysis was then repeated for six other Saccharomyces species, using the promoters for genes orthologous to the ones used in the S. cerevisiae analysis. As controls, we derived 20 gene sets whose members have predicted binding potential in S. cerevisiae that is closely matched to the bound and regulated genes. As expected, there is dramatically greater conservation of binding potential for the Leu3 targets than for the control sequences in the most distantly related species (Fig 2A).

**Figure 3:**
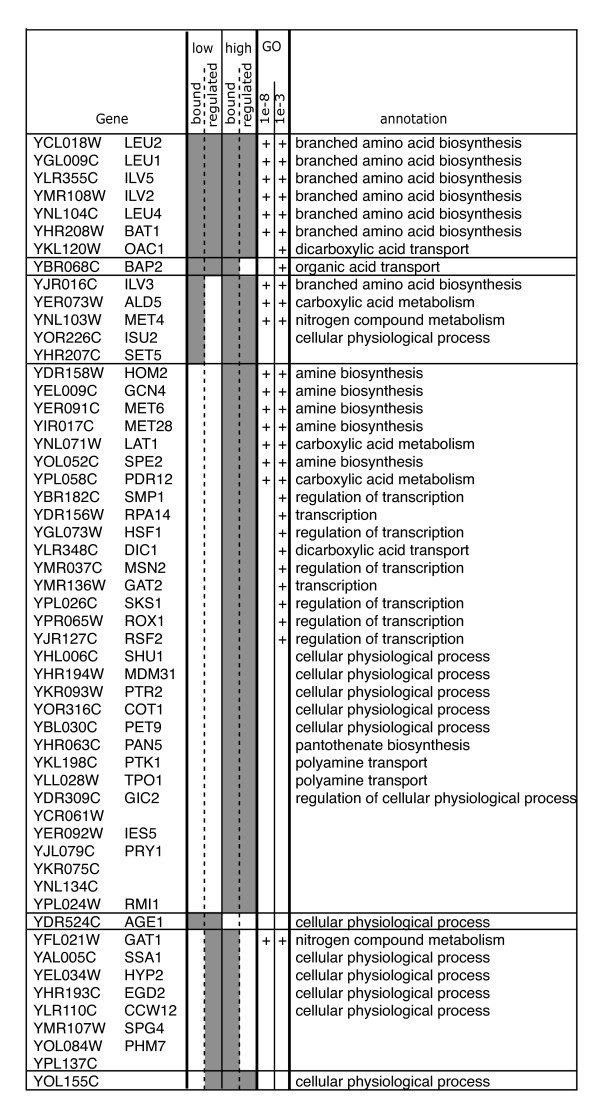
**Leu3 targets inferred from at least one combination of ChIP and expression analyses**. Genes are grouped according to the combinations of experiments that support the identification of the gene as a Leu3 target. Shaded portions of columns identify the genes that are significantly bound or regulated under the indicated experimental condition (low or high activity). The two columns labeled "GO" show the genes whose Gene Ontology process annotations are enriched at the indicated confidence levels (1e-3 or 1e-8). Annotations are shown for all genes found with GO annotations enriched with p-value better than 0.05. If more than one annotation was enriched, the most significant annotation is shown. The GO annotation "branched chain family amino acid biosynthesis" has been abbreviated to "branched amino acid biosynthesis".

We next performed this analysis on the genes identified as bound and regulated in the high-activity experiment. Excluding the nine genes that are identified using only the low-activity data, there are 45 genes identified using some combination of the high or low activity ChIP and expression data (Fig. 3). Fig 2B shows that the predicted binding potential of Leu3 for these new genes is generally better conserved than genes of similar predicted binding potential that are not bound and regulated. This reinforces the conclusion from GO enrichment analysis that the genes identified in the high activity experiment include novel direct targets that are biologically relevant.

### Metabolic functions of target genes imply an expanded physiological role for Leu3

There are 54 genes bound and regulated by Leu3 according to some combination of ChIP and expression experiments at low or high activity (Fig. 3). As this is many more than the number of genes involved in branched amino acid biosynthesis, GO analysis was performed on the full set of 54 genes to help understand the breadth of Leu3 function. Not surprisingly, the GO process enriched with the greatest confidence is "branched chain family amino acid biosynthesis" (7 genes; p = 9e-12). However, there are even larger sets of enriched categories, such as amine biosynthesis (12 genes; p = 2e-11) and carboxylic acid metabolism (15 genes; p = 3e-9). Altogether, there are 17 genes that have GO process annotations with P-values for enrichment of less than 1e-8, and there are 28 genes with GO processes that are enriched with more moderate confidence (p ≤ 1e-3). (Fig. 3).

**Figure 4:**
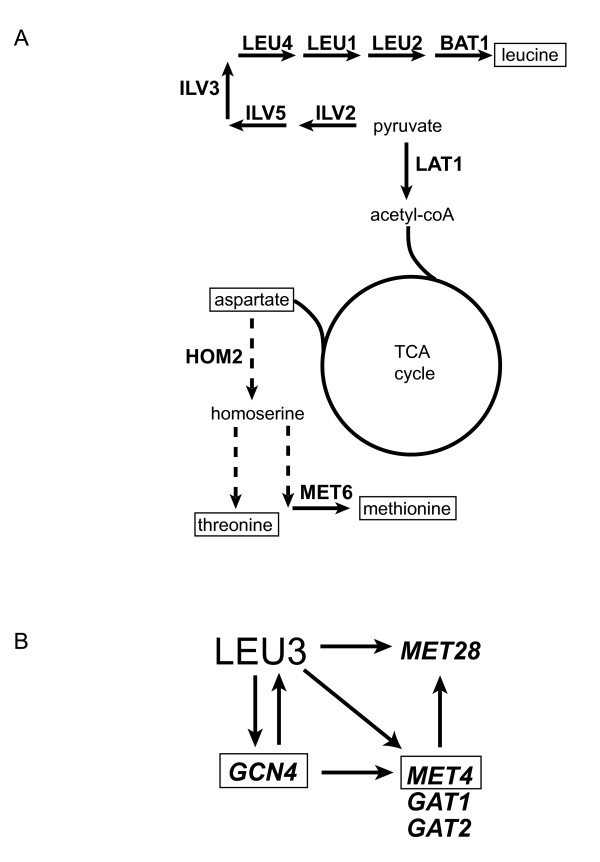
**Function of selected Leu3 target genes**. (A) Metabolic pathways in which Leu3 target genes function. Genes that are bound and regulated according to at least one combination of low or high activity expression and low or high ChIP analysis were subjected to GO process analysis, yielding 17 genes with enriched annotations. Gene names shown here are for those genes encoding enzymes directly involved in amino acid biosynthesis. (B) Transcriptional regulatory network involving Leu3. Interactions of Leu3 with other transcription factor genes are inferred from some combination of expression and ChIP experiments as described in the text. Interactions of transcription factors other than Leu3 are inferred from the literature.

The functions of some of the 17 bound and regulated genes that share highly enriched GO process annotations are shown in Fig 4A. This set includes genes for every enzymatic step on the committed pathway to leucine and valine synthesis, as well as three other genes that lead to the synthesis of other amino acids. GO-enriched genes not represented on this map consist of additional metabolic enzymes (SPE2, ALD5), a plasma membrane transporter (PDR12), and several transcription factors (MET4, MET28, GCN4, GAT1). These experiments and analyses imply a broader role for Leu3 in cellular physiology than the regulation of branched amino acid biosynthesis that is traditionally ascribed to this transcription factor.

### A transcriptional regulatory network defined by Leu3 targets

In addition to analyzing Gene Ontology process annotations, we also analyzed GO "function" annotations among the 54 bound and regulated genes. Remarkably, the three most significant annotations are related to transcriptional regulation, with a total of 10 genes annotated as having transcriptional regulator activity. The next most significant annotation is "organic acid transport" with four genes. The abundance of transcription factor genes among the bound and regulated targets of Leu3 is unexpected as Leu3 had previously been thought to function as a simple regulator of branched amino acid biosynthesis. However, some of the transcription factor target genes are consistent with a broader metabolic role for Leu3. Especially relevant is the observation that GCN4 appears to be a direct target of Leu3. GCN4 is the master regulator of general amino acid control and regulates LEU3 expression among many other targets [7,11,12]. Caution is in order as GCN4 could be one of the 9 or 10 genes that are expected to be in the intersection of the bound and expressed genes by chance, and we are unaware of any other evidence for regulation of GCN4 by Leu3. Nevertheless, our data suggest a positive feedback loop between Leu3 and Gcn4. Such a feedback loop makes physiological sense because leucine and valine together comprise about 15% of the amino acid residues in proteins, and starvation for branched amino acids could be a general signal for amino acid starvation. Met4 and Met28, which function together to control sulfur and sulfur amino acid metabolism, are also targets of Leu3, and binding to the former has been observed at endogenous concentrations as well [13]. Interestingly, there are a number of Leu3 target genes that have previously been shown to be bound as well by Gcn4 or Met4 [7]. These interactions, summarized in Fig 4B, suggest that Leu3 activates some genes through a feed-forward mechanism in which it both directly controls expression of a target gene as well as activating expression of a different transcription factor that targets the same gene.

Among the transcription factors that appear to be bound and regulated by Leu3 are three that are involved in stress response: HSF1 (heat shock response), MSN2 (binds to stress response elements), and SMP1 (osmotic stress). Since all three of these genes were identified as Leu3 targets only in the high concentration experiments, it is possible that stress is caused by elevated Leu3 activity itself. However, even if metabolic stresses play a role in induction, most of these genes appear to be regulated directly since they are bound by Leu3 as well as being induced.

### Utility of low and high activity perturbations

**Figure 5:**
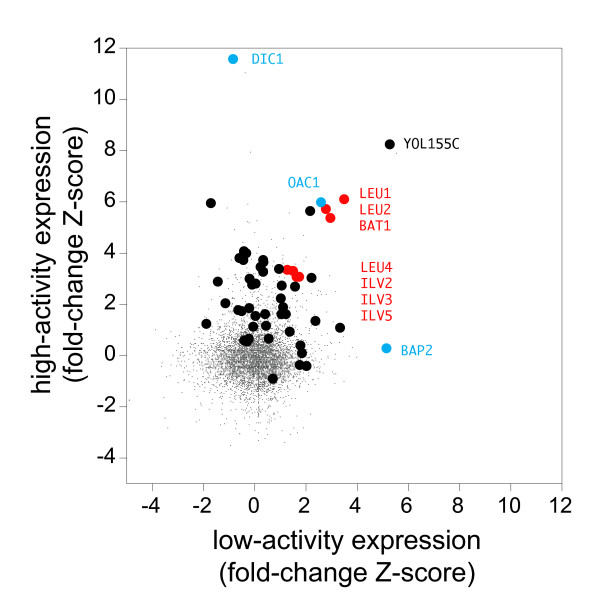
**Relative expression levels of bound genes in low and high activity experiments**. Fold-changes in expression levels were normalized to the standard deviation so that a Z-value of 1 corresponds to one standard deviation. Small points show the values for the 5000+ genes analyzed in each experiment. Large dots represent the 54 genes that are significantly bound and expressed according to some combination of low and high activity expression and ChIP data. Note that an estimate of statistical significance was used to identify differentially expressed genes, not the fold-change Z-value shown here. Gene and labels shown in red are the seven genes on the committed pathway to leucine and valine biosynthesis. Genes and labels shown in blue are annotated as organic acid transporters and are described in the text. The label "YOL155C" indicates a gene that is bound by Leu3 and is exceptionally strongly affected at both low and high activities, but which has no known function.

The expression levels of nearly all the classically defined targets of Leu3 are affected by both LEU3 deletion and Leu3 over-expression. Indeed, the seven genes that comprise the pathway for branched amino acid biosynthesis are among the most strongly regulated genes under each condition (Fig 5). This suggests that the primary physiological targets can largely be identified from either deletion of the transcription factor or its overexpression. On the other hand, GO analysis and the conservation of predicted binding potential both suggest that authentic target genes can be responsive to only one of the perturbations. This is illustrated well by a set of permeases and transport proteins that are bound and regulated by Leu3. As noted above, "organic acid transport" is the second most significant functional annotation among the 54 genes that are bound and regulated (p < 4e-4), with a total for four genes represented (BAP2, PDR12, OAC1, DIC1). BAP2 (leucine-specific permease) is a well-known target of Leu3 and its expression is strongly affected by Leu3 deletion. However, we found here that BAP2 expression is not induced by elevated Leu3 activity. In contrast, DIC1 (mitochondrial dicarboxylate carrier). Is very strongly affected by high Leu3 activity, but is not affected by Leu3 deletion. The other two transport proteins are affected by both perturbations. PDR12 is only modestly affected, but OAC1 (mitochondrial oxaloacetate carrier) is strongly affected. Indeed, its expression changes under both conditions are very similar to the leucine biosynthetic enzyme genes LEU1, LEU2 and BAT1 (Fig 5).

These examples illustrate the conclusions that we have drawn from the analysis of complete gene sets described above. To summarize, the combination of low activity ChIP and expression experiments identify most of the genes that are the primary physiological targets of Leu3. However, high activity ChIP and expression experiments, either by themselves or in combination with the low activity experiments, identify additional targets. These targets are enriched in authentic biologically relevant targets, as judged by GO annotation analysis and by conservation of predicted binding potential.

## Conclusion

Expression analyses typically identify a large number of differentially regulated genes, but most such experiments involve systems or perturbations that are inherently more complex than what we have studied here as a model system. The Leu3 regulon is exceptionally simple by most standards, and the perturbations we have made are arguably the most direct perturbations possible. Nevertheless, we find that 10% of all yeast genes have expression levels that are affected by these perturbations, and that almost all of these effects are indirect rather than being due to binding by Leu3. This conclusion is based on the failure to detect binding of Leu3, combined with a computational analysis that shows that most of the unbound genes are not expected to be bound (that is, do not have predicted Leu3 binding potentials that are higher than unregulated genes).

Sensitivity of the ChIP assay does seem to be a factor in the failure to identify some true targets. The importance of the high-concentration ChIP analysis is that it gives a sense for what is missing from ChIP analyses performed at endogenous concentrations. At one extreme are genes that are nearly fully occupied at low concentration. Occupancy of these genes is effectively saturated and will not appear to be substantially more bound at higher concentrations. At the other extreme, genes that are bound with vanishingly low occupancy at low concentrations will remain undetected at high concentrations because the probability of binding is still very low. In between these extremes, however, are the genes that are bound at low concentration but fall below the detection threshold. It is these genes that can be revealed by performing ChIP experiments at higher-than-endogenous concentrations. We note that some of the genes that are identified as being novel Leu3 targets arise from the intersection of the high activity ChIP data with the low activity expression data. That is, these genes are detectably bound only at high concentrations, but the only significant effect on expression is observed when comparing wild-type cells with a leu3 deletion. Our interpretation is that these genes are bound by Leu3 at endogenous levels, but not sufficiently well to be detected in the ChIP experiment. Thus, the failure to detect binding can be attributed to inadequate sensitivity of the ChIP assay for at least some true target genes. For most of the genes we call indirect targets, however, failure to detect binding cannot be attributed to low ChIP sensitivity because the predicted binding potential of these genes is no higher than that of average gene in the genome.

Direct transcription factor perturbation by deletion or overexpression is much simpler than most perturbations. In the case of environmental perturbants, for example, it may not even be known how many transcription factors are involved or how the effect of the perturbation is mediated. Nevertheless, it is clear from the analyses presented here that even direct perturbation produces a large number of effects that are not directly related to binding of the transcription factor. Additional experimental methods, such as double mutant analyses, may help to elucidate networks of direct transcriptional control [14]. Also required are innovative computational methods for combining information from expression and ChIP experiments [15,16].

## Methods

### Strains and growth conditions

The LEU3 gene, containing a mutation that confers constitutive activation activity on Leu3, was fused to the gene for maltose binding protein on a URA3-containing 2 micron-based plasmid, and transformed into BY4720-leu3Δ^neo^. As a reference strain for expression experiments, we also transformed into BY4720-leu3Δ^neo ^a plasmid expressing only the MBP-tagged DNA binding domain of Leu3 (i.e., missing sequences needed for transcription activation). (XL, Cheol-Koo Lee, Joshua A. Granek, NDC, and Jason D. Lieb; submitted). Fusion proteins were expressed from the constitutively active TDH3 promoter. Cells were grown at 30°C in media lacking uracil to maintain selection for the plasmid.

### Expression analysis

The same strain used for the chromatin immunoprecipitation experiments was used for Affymetrix-based transcriptome analysis, and the level of expression of each gene was compared to that of the BY4720-leu3Δ^neo ^parent strain carrying a plasmid expressing the Leu3 DNA binding domain only. The cells were cultured in uracil dropout medium (YNB-AA (Sigma) 6.7 g/L, 0.77 g/L, Ura DO Supp. (BD Bioscience, Palo Alto, CA), and 2% glucose supplemented with G418 at 200 mg/L) with shaking at 30°C. The cells were harvested in log-phase growth (A_600 _~ 0.7). The total RNA was isolated using RNeasy mini kit (Qiagen, Valancia, CA), following the enzymatic lysis protocol suggested by the manufacture. Briefly, a total of less than 5 × 10^7 ^cells were harvested by spinning at 1000 × g for 5 min at 4°C. The cells were resuspended in 2 ml of lysis buffer Y1 (1 M sorbitol, 0.1 M EDTA, pH 7.4, with 0.1% β-mercaptoethanol and 10 U yeast lytic enzyme (MP Biomedicals, Aurora, Ohio)/1 × 10^7 ^cells) added just before use. The mixture was gently shaken at 30°C for 10 min, followed by centrifugation at 300 g for 5 min. 350 μl Buffer RLT was added to the pellet and vortexed vigorously. 350 μl 70% ethanol was then added, and the total sample was transferred to the RNeasy mini column. The column was sequentially washed with 700 μl Buffer RW1, then twice with 500 μl Buffer RPE, followed by centrifugation at greater than 8000 g for 15 seconds Total RNA was collected by eluting at 8000 g for 1 min with 50 μl RNase-free water. Four biological replicates were analyzed for each strain. Total RNA was sent to the Microarray facility of Johns Hopkins University for labeling and hybridization to the Affymetrix chip YG_S98.

The R statistical packages, *rma and affy*, were used for background correction, data normalization and estimation of p-values for differential expression [17,18]. Benjamini and Hochberg procedures was also applied for control of the false discovery rate (FDR). There are a total of 9,335 probe sets on the YG_S98 chip, but only 5,592 sets that correspond to protein coding transcripts for which we calculated binding probabilities with GOMER [4]. This reduced set of probes was used for subsequent analyses.

### Chromatin immunoprecipitation

The chromatin immunoprecipitation experiments have been submitted elsewhere in the context of an analysis of chromatin effects on binding site selection (XL, Cheol-Koo Lee, Joshua A. Granek, NDC, and Jason D. Lieb; submitted). Enrichment was detected using a microarray that covers nearly all of the yeast genome, with most of the spots corresponding either to ORFs or to intergenic regions [1]. For the analyses in this paper, genes were ranked based on the product of two ChiP enrichment p-values [19], one corresponding to enrichment of the coding sequence and one corresponding to its promoter. In those cases where there are two or more microarray features overlapping the 600 bp 5' to an ORF, the microarray feature with the lowest p-value was used to represent promoter binding. Enrichment of ORF features was factored into the ranking of genes because preliminary analyses showed that genes that were bound in their promoters and regulated were also enriched in apparent binding to ORFs. While this can be an artifact due to promoter-bound sequences overlapping ORF sequences on the array [1,8], we found that ORFs that were bound and induced were significantly enriched in binding sites relative to unbound ORFs. Thus, detection of binding to ORFs among bound and regulated genes is not entirely an artifact of binding to adjacent promoters in this case.

### Selection of genes bound and regulated at high concentration

Direct comparison of ChIP and expression experiments is problematic because both the low and high activity ChIP experiments were performed with spotted PCR product arrays while both the low and high activity expression experiments were performed using Affymetrix oligonucleotide arrays. For the low activity data we chose to use threshold values provided by the authors of those studies [6,7]. For our own data, we chose to define threshold values for binding and induction that maximize the fraction of genes that are both bound and induced, above and beyond the number that is expected by chance. Specifically, the top *B *bound genes (50, 100, 150 ...) were compared to the top *I *induced genes (50, 100, 150, ...), and for each combination we determined the value of ((B ∩ I) - (B•I/T))/(B+I), where T is the total number of genes for which there is both expression and binding data. Applying this standard to find the maximal significant overlap, we found 44 genes in common among the top 200 bound genes and the top 250 induced genes. Compared to the number of genes that meet each individual criterion, the 44 genes that are bound and regulated is significantly greater than the 9 or 10 expected by chance (p = 4e-20). A list of the bound genes, regulated genes, and genes that are both bound and regulated by this criterion is provided as Additional file 1.

### Calculation of predicted binding potential and assessment of conservation

The program GOMER was used to calculate the Leu3 binding potential for all sequences within 600 bp upstream of an ORF, and to compare the potential of genes in an input list (e.g., bound and regulated genes) to other genes in the genome [8]. The binding potential score calculated by GOMER reflects all of the potential binding sites in the regulatory region. The promoters of orthologous genes were scored in an identical manner. Genes that show unusually well conserved potential to bind Leu3 typically have binding sites that are themselves orthologous. However, the algorithm does not require that this be the case as no alignment of the promoter regions is involved. Independently evolved binding sites can contribute to the binding potential score, and to the conservation of this score. The position weight matrix used in these calculations was based on the measurement of Kd values for 50 different Leu3 binding site variants [9]. We also performed a motif discovery analysis using the genes that are regulated but not bound under high activity conditions. BioProspector was run on the 600 bp upstream of the regulated genes [20]. Default were used except that widths of 6, 8 10 and 12 were used rather than just the default of 10 bp. Sets of upstream sequences that contained the same number of genes as the experimental set were randomly selected and used as controls to assess significance.

## Authors' contributions

LT performed and analyzed the expression experiments and was principally responsible for the integration of expression and ChIP data. XL initiated the conservation analysis and helped integrate ChIP and expression analyses. NDC helped design the experiments, was involved in all analyses, and wrote the paper. All authors have helped revise the paper and have read and approved the final manuscript.

## Supplementary Material

Additional file 1 Bound, regulated and bound+regulated genes at high activity.txt. Tab delimited text file listing the 200 bound genes, 250 regulated gene, and 44 bound and regulated genes determined at high activity and defined as described in the text. Click here for file
